# Supplementary material for: Cultural determinants of the gap between self-estimated navigation ability and wayfinding performance: evidence from 46 countries
Source: Sci Rep. 2023 Jul 5;13:10844. doi: 10.1038/s41598-023-30937-w (PMC10322842; doi:10.1038/s41598-023-30937-w)
Supplement: Supplementary file 1 — Supplementary Information. [file 41598_2023_30937_MOESM1_ESM.pdf]

Supplementary Information for

Cultural determinants of the gap between self-estimated navigation ability and wayfinding performance: evidence from 46 countries.

Appendix A - Tables and Figures

Table S1. Descriptive statistics by country and gender for the sample of 383,187 players representing 46 countries used in this study.

					Females			Males		
	Country	N	Mean age	SD age	N	Mean age	SD age	N	Mean age	SD age
1	Argentina	1774	33.87	12.61	671	33.92	13.44	1103	33.83	12.08
2	Australia	7952	39.29	14.36	4031	39.35	14.98	3921	39.22	13.69
3	Austria	1974	36.66	13.51	809	34.98	14.19	1165	37.83	12.89
4	Belgium	1941	38.6	14.56	778	39.24	15.13	1163	38.17	14.16
5	Brazil	6105	32.66	12.4	2676	32.28	13.27	3429	32.96	11.67
6	Canada	10851	40.19	14.77	5187	39.99	15.45	5664	40.36	14.11
7	Chile	1606	33.61	12.68	640	31.88	12.93	966	34.76	12.38
8	China	5189	27.68	7.53	2580	26.94	7.23	2609	28.42	7.74
9	Colombia	1457	31.04	11	551	30.63	11.57	906	31.3	10.63
10	Costa Rica	596	34.16	12.24	256	33.83	13.18	340	34.41	11.48
11	Czech Republic	17023	29.9	10.6	7958	29.33	10.99	9065	30.4	10.22
12	Denmark	1714	37.08	13.12	585	35.88	13.8	1129	37.7	12.71
13	Finland	704	35.84	12.25	269	35.06	12.8	435	36.33	11.88
14	France	7943	36.74	13.94	3285	36.8	14.94	4658	36.69	13.19
15	Germany	34495	40.71	13.51	15632	40.15	13.9	18863	41.18	13.15
16	Greece	23745	32.84	10.59	9355	31.83	10.73	14390	33.49	10.44
17	Hong Kong	989	34.12	11.97	379	31.41	11.48	610	35.8	11.96
18	Hungary	10728	31.21	11.22	4748	30.52	11.41	5980	31.76	11.03
19	India	3341	26.73	8.14	496	29.13	10.65	2845	26.31	7.54
20	Indonesia	1095	27.3	8.47	330	26.23	7.54	765	27.76	8.8
21	Ireland	1408	39.43	13.13	546	39.01	14.36	862	39.7	12.29
22	Islamic Republic of Iran	1071	31.99	8.97	281	31.04	9.64	790	32.32	8.7
23	Italy	11780	35.39	13.56	4573	34.2	13.75	7207	36.14	13.38
24	Malaysia	1161	29.04	10.48	444	28.14	10.16	717	29.6	10.64
25	Mexico	3923	29.51	10.73	1492	28.64	10.74	2431	30.04	10.69
26	Netherlands	22877	35.97	13.43	12490	35.9	13.75	10387	36.05	13.04
27	New Zealand	1700	40.07	14.76	939	40.23	15.44	761	39.88	13.88
28	Norway	1140	35.25	12.33	483	32.79	12.13	657	37.05	12.17
29	Philippines	1370	27.86	9.88	673	26.8	9.19	697	28.88	10.4
30	Poland	9209	29.34	9.73	4496	28.52	9.89	4713	30.13	9.51
31	Portugal	2077	34.65	11.02	850	32.89	11.14	1227	35.86	10.78
32	Romania	3208	29.82	9.11	1144	28.1	8.91	2064	30.78	9.08
33	Russian Federation	2761	27.58	7.98	948	26.18	8.24	1813	28.32	7.74
34	Singapore	1338	32.48	11.49	529	31.22	11.18	809	33.3	11.62
35	Slovakia	5096	29.31	10.14	2297	28.7	10.28	2799	29.81	10
36	South Africa	1126	37.92	13.56	455	38.58	14.24	671	37.47	13.07
37	Spain	6797	36.99	13	2661	36.43	13.46	4136	37.36	12.68
38	Sweden	1793	35.85	12.72	667	34.55	12.88	1126	36.62	12.56
39	Switzerland	3136	41.82	14.1	1330	40.51	14.78	1806	42.78	13.51
40	Taiwan	1511	32.34	10.72	634	31.71	11.45	877	32.8	10.15
41	Thailand	871	30.88	11.67	322	28.83	10.62	549	32.08	12.1
42	Turkey	1835	31.19	9.75	602	29.35	9.61	1233	32.09	9.69
43	Ukraine	647	27.59	8.29	181	26.99	8.53	466	27.83	8.2
44	United Arab Emirates	685	35.68	11.1	197	33.92	11.6	488	36.38	10.82
45	United Kingdom	66196	42.94	14.11	28727	42.68	14.73	37469	43.14	13.61
46	United States	87249	38.99	14.55	43792	39.77	15.15	43457	38.21	13.89

Table S2. Left - odds ratios and t-values (along with p-values) for the multinomial logistic regression model with the self-estimated navigation skills (categorical) as the dependent variable and age band (categorical) as well as gender (categorical) as the independent variables. Right - probabilities of self-estimated navigation skills by gender and age band obtained from the logistic regression model.

Coefficients	Odds Ratios	t value
**Predictors:**		
Age band (reference level: *19-29*)		
30-39	1.22	22.89***
40-49	1.30	29.24***
50-59	1.30	26.30***
60-70	1.12	8.97***
Gender (reference level: *female*)		
male	2.61	145.16***
**Intercepts:**		
very bad | bad	0.03	-264.99***
bad | good	0.24	-209.45***
good | very good	4.03	208.41***
R^2 Nagelkerke	0.070	

*p < 0.05; **p < 0.01; ***p < 0.001.

			Probabilities of self-estimated navigation skills			
	Gender	Age band	very bad	bad	good	very good
1	female	19-29	0.0291	0.1658	0.6062	0.1989
2	male	19-29	0.0113	0.0736	0.522	0.3931
3	female	30-39	0.024	0.142	0.6021	0.2318
4	male	30-39	0.0094	0.0616	0.4886	0.4405
5	female	40-49	0.0224	0.1341	0.5988	0.2447
6	male	40-49	0.0087	0.0577	0.4756	0.458
7	female	50-59	0.0226	0.1346	0.5991	0.2437
8	male	50-59	0.0088	0.058	0.4766	0.4567
9	female	60-70	0.0261	0.1522	0.6048	0.2168
10	male	60-70	0.0102	0.0666	0.5038	0.4194

Table S3. Hierarchical regression analysis of predictors of wayfinding performance. Model 1 (without the self-estimates of navigation skills) is significantly weaker in terms of explained variance than Model 2 (with the self-estimates of navigation skills). Model 2 shows that the self-estimated navigation skills variable is a significant predictor of wayfinding performance measured across six wayfinding tasks (SHQ game levels: 3, 6, 7, 8, 11, and 12).

Predictor variables	Model 1		Model 2	
	standardized ß coefficient	*t* value	standardized ß coefficient	*t* value
Intercept	-0.399	40.544***	-0.567	38.388***
Age	-0.352	232.692***	-0.355	234.788***
Gender (reference level: *female*)				
male	0.462	-153.757***	0.433	-140.304***
Education (reference level: *no formal*)				
high school	0.105	-10.976***	0.099	-10.409***
college	0.187	-19.658***	0.179	-18.928***
university	0.207	-22.211***	0.199	-21.339***
Travel time (reference level: *up to 30 mins*)				
30 mins to 1 hour	-0.008	2.276*	-0.012	3.555***
1 hour+	-0.030	7.708***	-0.042	10.949***
Home environment (reference level: *rural*)				
mixed	0.028	-6.953***	0.031	-7.749***
city	-0.090	20.230***	-0.086	19.394***
Navigation skills (reference level: *very bad*)				
bad	-	-	0.078	-6.331***
good	-	-	0.178	-15.364***
very good	-	-	0.263	-22.440***
R^2	0.1789		0.1824	
R^2 change	-		0.0035	

*p < 0.05; **p < 0.01; ***p < 0.001.

Table S4. ANOVA tables of terms in the linear regression models without self-estimates of navigation skills (Left), with self-estimates of navigation skills (Right), and for the F-test of differences between both models (Bottom).

	Sum Sq	Df	F value	Pr(>F)
Age	44332.08	1	54145.661	0
Gender	19356.316	1	23641.131	0
Education	944.064	3	384.349	0
Travel time	50.244	2	30.683	0
Home environment	974.059	2	594.841	0
Residuals	303401.465	370564		

	Sum Sq	Df	F value	Pr(>F)
Age	44939.321	1	55125.378	0
Gender	16047.676	1	19685.082	0
Education	883.151	3	361.109	0
Travel time	99.894	2	61.268	0
Home environment	957.642	2	587.352	0
Navigation skills	1312.654	3	536.728	0
Residuals	302088.811	370561		

	Res.Df	RSS	Df	Sum of Sq	F	Pr(>F)
1	370564	303401.465				
2	370561	302088.811	3	1312.654	536.728	0

Table S5. Proportions of self-reported navigation skills in the sample by country.

		Proportions of self-estimated navigation skills			
	Country	very bad	bad	good	very good
1	Argentina	1.7%	8.4%	49.3%	40.6%
2	Australia	2.1%	11.7%	56.4%	29.8%
3	Austria	0.6%	3.8%	37.9%	57.7%
4	Belgium	2.4%	11.9%	58.9%	26.8%
5	Brazil	2.3%	10.9%	53.4%	33.3%
6	Canada	2.0%	11.5%	52.9%	33.7%
7	Chile	1.5%	9.3%	52.5%	36.7%
8	China	3.9%	16.4%	57.3%	22.4%
9	Colombia	1.3%	10.1%	54.2%	34.4%
10	Costa Rica	2.2%	9.2%	49.0%	39.6%
11	Czech Republic	0.8%	7.6%	59.3%	32.3%
12	Denmark	2.4%	11.0%	53.2%	33.4%
13	Finland	2%	13%	60%	25%
14	France	2%	14%	59%	24%
15	Germany	0.3%	3.4%	42.8%	53.4%
16	Greece	1.7%	10.3%	51.6%	36.4%
17	Hong Kong	2%	15%	55%	27%
18	Hungary	1.9%	10.5%	59.0%	28.6%
19	India	1.9%	10.9%	58.7%	28.6%
20	Indonesia	1%	12%	60%	27%
21	Iran	2%	14%	57%	26%
22	Ireland	1.7%	9.8%	56.5%	32.0%
23	Italy	2%	12%	55%	31%
24	Malaysia	2.4%	15.5%	61.0%	21.1%
25	Mexico	1.3%	9.6%	55.8%	33.3%
26	Netherlands	2.0%	14.5%	62.7%	20.8%
27	New Zealand	1.9%	10.7%	58.9%	28.5%
28	Norway	2%	12%	60%	26%
29	Philippines	1.2%	10.8%	63.0%	25.0%
30	Poland	1.1%	9.4%	56.8%	32.7%
31	Portugal	2.3%	11.6%	59.1%	27.1%
32	Romania	1.0%	6.9%	59.8%	32.3%
33	Russian Federation	1.4%	10.5%	54.2%	33.9%
34	Singapore	3%	14%	56%	26%
35	Slovakia	1.1%	6.2%	58.0%	34.8%
36	South Africa	2.3%	10.9%	51.6%	35.2%
37	Spain	2%	12%	55%	30%
38	Sweden	1.6%	11.6%	56.3%	30.4%
39	Switzerland	1.08%	6.82%	46.33%	45.76%
40	Taiwan	2.2%	16.4%	59.8%	21.6%
41	Thailand	2.4%	12.3%	60.3%	25.0%
42	Turkey	2%	13%	53%	33%
43	Ukraine	1%	12%	56%	30%
44	United Arab Emirates	2.6%	9.9%	48.0%	39.4%
45	United Kingdom	1.9%	11.3%	56.9%	29.8%
46	United States	2.0%	10.5%	53.0%	34.5%

Table S6. Sample size and proportions of self-reported navigation skills for each cultural cluster.

	Cultural cluster	N	Proportions of self-estimated navigation skills			
			very bad	bad	good	very good
1	Anglo	175356	2.0%	10.9%	54.7%	32.4%
2	Eastern Europe	48672	1.2%	8.6%	58.3%	31.9%
3	Germanic	39605	0.4%	3.7%	42.8%	53.0%
4	Latin Europe	30538	2%	13%	57%	29%
5	Nordic	28228	2.0%	14.0%	61.5%	22.5%
6	Near East	25580	1.7%	10.4%	51.7%	36.2%
7	Latin America	15461	1.8%	10.0%	53.4%	34.9%
8	Confucian Asia	9027	3.3%	16.0%	57.3%	23.4%
9	Far East	8909	2%	12%	60%	26%
10	African	1126	2.3%	10.9%	51.6%	35.2%
11	Arabic	685	2.6%	9.9%	48.0%	39.4%

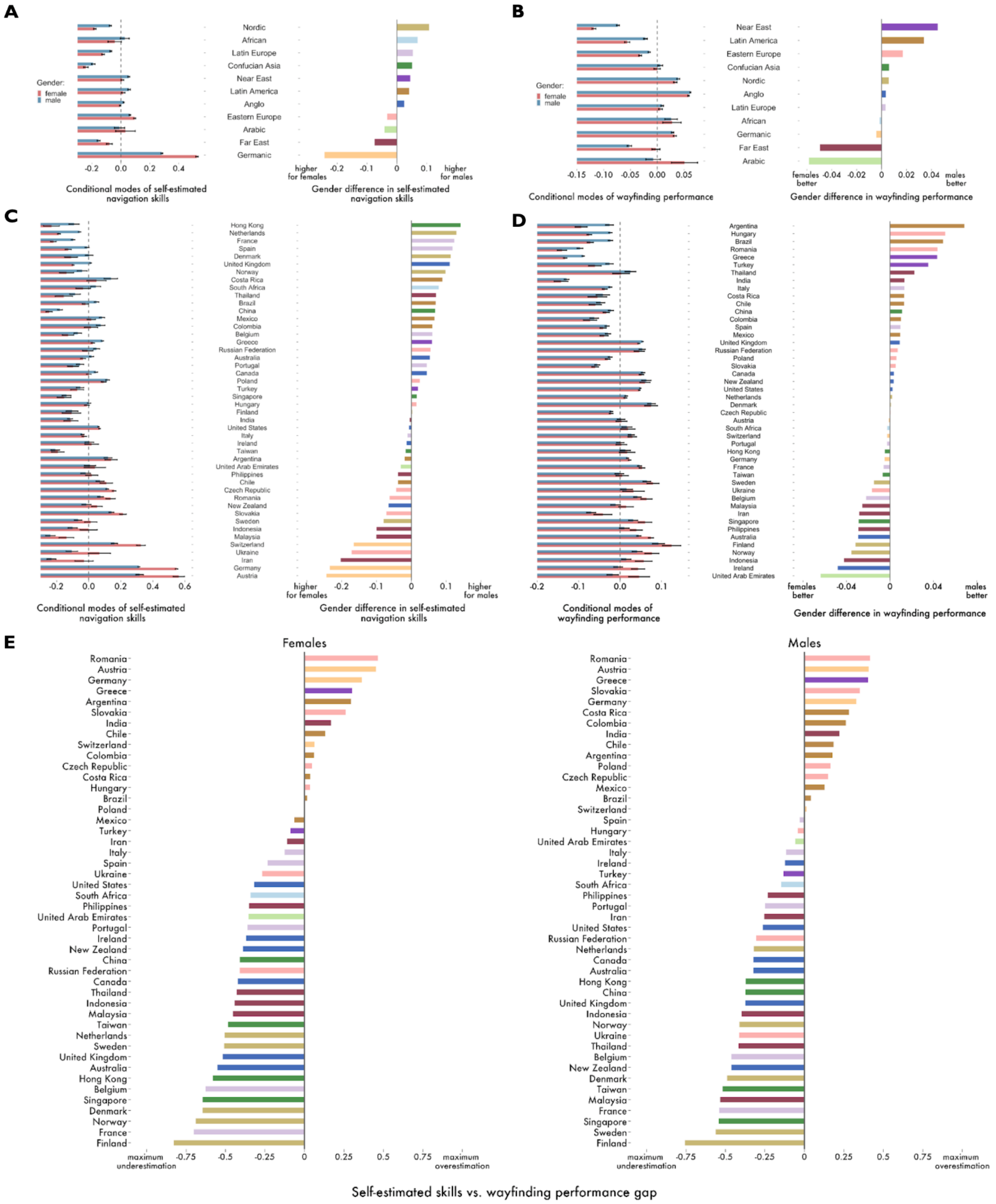

Figure S1. Self-estimated navigation skills, wayfinding performance and the gap between self-reported skills and actual performance by cultural cluster and country for females and males. A) Conditional modes self-estimated navigation skills by cultural cluster and gender. B) Conditional modes of wayfinding performance by cultural cluster and gender. C) Conditional modes self-estimated navigation skills by country and gender. D) Conditional modes of wayfinding performance by country and gender. E) The gap between self-estimated navigation skills and wayfinding performance by country for females and males.

Appendix B - Additional Results

The relationships between the self-estimated navigation skills vs. wayfinding performance gap for each country and Hofstede's cultural dimensions as well as global indices.

Self-estimated navigation skills vs. wayfinding performance gap by country: calculated as the difference between min-max normalised navigation skills (estimated as conditional modes obtained from a linear mixed model with self-reported ability as the dependent variable, controlled for age and gender, with varying intercepts for countries) and min-max normalised wayfinding performance (estimated as conditional modes obtained from a linear mixed model with wayfinding performance i.e. reversed MSCD metric as the dependent variable controlled for age and gender, with varying intercepts for countries); range of [-1, 1], where:

- values close to -1 denote 'high underestimation',
- values around 0 mean 'accurate estimation'
- and values closer to 1 translate to 'high overestimation'.

As the gap metric was not normally distributed and most of the global indices and Hofstede's cultural dimensions were also not normally distributed, we estimated the significance of all relationships reported in this appendix with the Spearman's *rho* test.

Correlations between the Self-Estimated Navigation Skills vs. Performance Gap and Hofstede's cultural dimensions:

- Power Distance: Spearman's *rho* = 0.11, p = .491
- Individualism: Spearman's *rho* = -0.18, *p* = .242
- **Masculinity: Spearman's *rho* = 0.43, p = .004****
- **Uncertainty Avoidance: Spearman's *rho* = 0.47, p = .001****
- Long-Term Orientation: Spearman's *rho* = -0.12, *p* = .448
- Indulgence: Spearman's *rho* = -0.18, *p* = .28

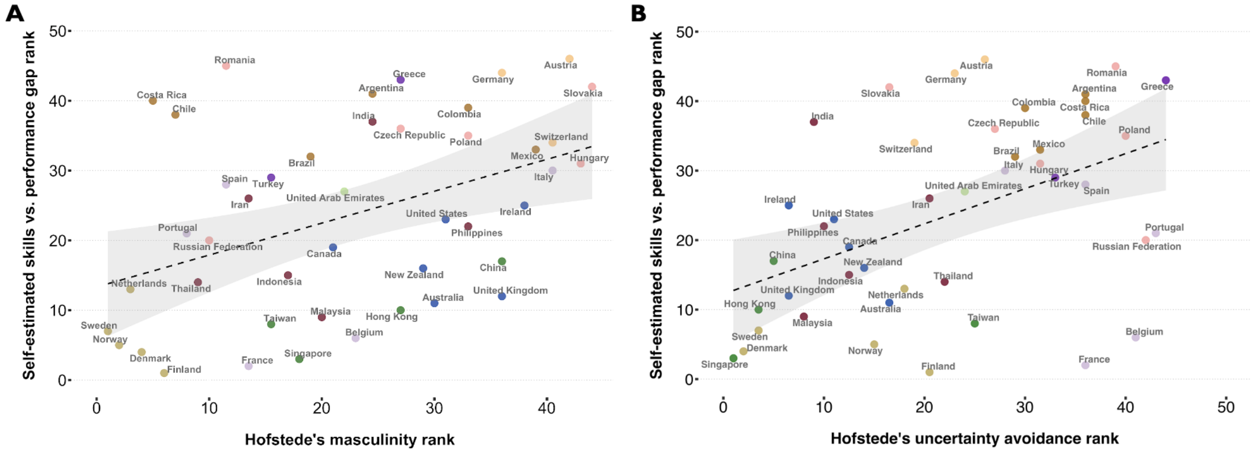

Figure S2. The Spearman's relationships between the ranks of the self-estimated navigation skills vs. performance gap and the ranks of Hofstede's dimensions of masculinity (A) and uncertainty avoidance (B).

The above six Hofstede's dimensions were further used in the linear regression model as independent variables of the self-estimated skills vs. performance gap (Table S7).

Table S7. The effects of Hofstede's cultural dimensions on the self-estimate-performance gap (standardised coefficients and their standard errors with asterisks denoting the level of significance - p-values Bonferroni adjusted). The model R^2=50.8% and adjusted R^2=42.6%.

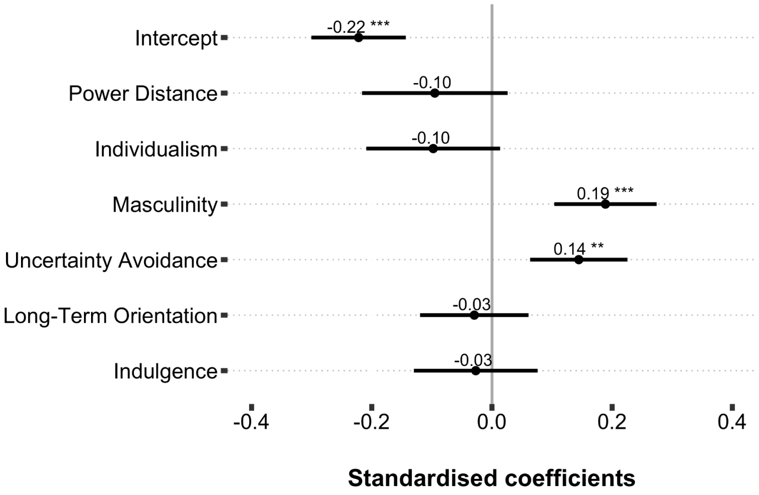

For completeness, we report other significant and non-significant correlations of the gap between the self-estimated navigation skills and wayfinding performance with the remaining global indices used in our study:

Correlation between the Self-Estimated Navigation Skills vs. Performance Gap and the Gender Inequality Index 2018:

- **Gender Inequality Index 2018: Spearman's *rho* = 0.40, *p* = .007****

Correlations between the Self-Estimated Navigation Skills vs. Performance Gap and the Global Gender Gap Report 2020:

- Global Gender Gap Index (overall): Spearman's *rho* = -0.23, *p* = .127
- **Economic Participation & Opportunity: Spearman's *rho* = -0.47, *p* = .001****
- Educational Attainment: Spearman's *rho* = -0.13, *p* = .399
- **Health & Survival: Spearman's *rho* = 0.37, *p* = .01***
- Political Empowerment: Spearman's *rho* = -0.06, *p* = .723

Correlation between the Self-Estimated Navigation Skills vs. Performance Gap and the GDP per capita (2019, World Bank):

- **GDP per capita (2019): Spearman's *rho* = -0.31, *p* = 0.044***

*p < 0.05; **p < 0.01; ***p < 0.001.
